# Reorganization

**Published:** 1900-05

**Authors:** 


### Reorganization.

The Colorado County Medical Association met in Weimar pursuant to adjournment on Tuesday, April 17th. The object of the meeting was chiefly to accomplish a reorganization of the society and place it upon a more permanent basis. Dr. W. T. McLeary, President, called the meeting to order, and, upon motion, Dr. J. F. Thornton was elected secretary pro tem., the secretary being absent on account of high water. Dr. T. C. Cook was elected vice-president to fill the unexpired term.

The reorganization of the society was perfected with a membership of about eighteen representatives of the medical profession, from different sections of the county—the Eagle Lake contingent being absent on account of the destruction of the railroad bridge across the Colorado river.

After the transaction of routine business—the chief item of which was the appointment of Dr. J. F. Hutchins as a delegate, and Dr. C. G. Cook alternate, to the State Medical Association, which is to assemble in Waco, Tuesday, 24th inst.—a preamble and resolutions were adopted calling attention to the insufficiency of our health laws, and pledging members of the Association to exert themselves to secure the election of such men to the Legislature and other State officers as were in favor of a thorough revision of the sanitary laws of the State. The Association adjourned to meet again in Columbus on the third Tuesday in May.

Colorado County Medical Association adopted the following resolutions:

WEIMAR, April, 17, 1900.

WHEREAS, Smallpox was introduced into Colorado county during the winter of 1898; and

WHEREAS, Our commissioners' court provided for the isolation of the cases thereof at an expense of more than $6000 to Colorado county, by which the disease was effectually stamped out; and

WHEREAS, Our neighboring counties having failed, refused, and neglected to take a similar precaution as in their discretion (which was legal) our county became reinfected, and there is now within its limits probably one hundred cases of this loathsome disease, while, according to the report of the Marine Hospital Service, there are more than 500 cases in the State at present, thus demonstrating the total insufficiency of our present health laws to protect the public health; therefore, be it

*Resolved,* That we, as an association, will use all of our influence to secure the election of members to both branches of the Legislature, and all other State offices, who are in hearty accord with the unselfish recommendations of the State Medical Association to thoroughly revise our State health laws and place them in front rank on this subject with the civilization of the day.

*Resolved further,* That our delegate to the State Medical Association be and is hereby instructed to urge this sentiment before that honorable body at its ensuing session at Waco, Texas.

W. T. MCLEARY, President.

J. F. THORNTON, Secretary.

---

The Brazos Valley Medical Association held a large and interesting meeting at Caldwell, Texas, May 8th and 9th inst. An attractive program was carried out. We hope to present our readers some of the papers read on the occasion. It is to be regretted that the JOURNAL did not receive the program in time for publication in April, and the meeting was over before our May issue appeared. The Brazos Valley Medical Association is flourishing.

